# Supplementary material for: A combined association of serum uric acid, alanine aminotransferase and waist circumference with non-alcoholic fatty liver disease: a community-based study
Source: PeerJ. 2022 Mar 4;10:e13022. doi: 10.7717/peerj.13022 (PMC8900609; doi:10.7717/peerj.13022)
Supplement: Supplemental Information 1 [file peerj-10-13022-s001.pdf]

识别编码：|__|__|__|__|__|__|__|__|__|__|__|__|__|__|__|__|__|__|

健康调查问卷

您好！

您好！这是一份健康调查问卷，目的是通过调查了解您的健康状况，请您根据自身实际状况填写答案。本调查所得数据用于您的健康体检数据分析，您的个人信息我们将做好保密工作。恳请您能协助完成本次调查。感谢您的支持与配合！

圈选出最符合的选项或根据问题在横线上填写答案。

一、基本信息

A1.您的性别：1…男　　2…女

A2.您的出生日期：______年___月___日

A3.您的民族：

1…汉族　　2…非汉族（请在横线上注明：_____）

A4.您本人的最高文化程度是什么？

1…小学　　2…初中　　3…高中（中专/技校）　　4…大专　　5…本科

6…硕士　　7…博士及以上

A5.您目前的婚姻状况是：

1…未婚　　2…已婚　　3…离异　　4…丧偶

二、吸烟、饮酒情况

S1.您过去经常吸烟（指每天吸烟 1 支以上，连续 6 个月及以上）吗？

0…否　　1…是

S2.如果是，大多数情况下您每天吸烟___支。

AL1.您过去经常饮酒（指每周饮酒 3 次以上，连续 6 个月及以上）吗？

0…否　　1…是

AL2.如果是，大多数情况下您平均每天饮酒___毫升。

AL3.大多数情况下您的饮酒种类为？

1…白酒　　2…啤酒　　3…红酒　　4…葡萄酒　　5…洋酒

6…其他（请在横线上注明：______）

三、个人疾病史及家族史

<u>请您根据实际情况将相应选项的数字编号填写到对应的格子里。</u>

病种	您是否曾被医生诊断过相关疾病？<br>0…否 1…是 9…不记得	确诊年份	您的直系亲属是否曾被医生诊断过相关疾病？<br>0…否 1…是 9…不清楚	与您的关系<br>1…父亲 2…母亲 3…兄弟或姐妹 4…子女
高血压病				
糖尿病				
高脂血症				
脂肪肝				
肝硬化				
病毒性肝炎				
冠心病				
胆囊炎				
脑卒中				
恶性肿瘤				
精神疾病				
