# Supplementary material for: A combined association of serum uric acid, alanine aminotransferase and waist circumference with non-alcoholic fatty liver disease: a community-based study
Source: PeerJ. 2022 Mar 4;10:e13022. doi: 10.7717/peerj.13022 (PMC8900609; doi:10.7717/peerj.13022)
Supplement: Supplemental Information 2 [file peerj-10-13022-s002.pdf]

Identification code: |__|__|__|__|__|__|__|__|__|__|__|__|__|__|__|__|__|__|

Questionnaire for health survey

Hello!

Hello! This is a questionnaire for investigating your health condition. Please put the answer according to your actual situation. This survey data will only be used for healthy analysis, and will be kept confidential. Please help us to complete the survey, thank you for your support and cooperation!

Circle the answer best fitting you or write the answer on the line

Basic information

A1. Your gender: 1···male   2···female

A2. Your birthday: ______year___month___day

A3. Your nation:

1···the Han nationality   2···non-Han nationality (Please indicate on the line:______)

A4. What is your highest education level?

1···primary school   2···junior high school   3···senior high school (technical secondary school/ technical school)   4···junior college   5···undergraduate course   6···master   7···doctor or above

A5. What is your marital status?

1···single   2···married   3···divorced   4···widowed

Smoking and drinking

S1. Have you used to smoke? (>1 cigarette per day for 6 consecutive months or more)

0···no   1···yes

S2. If yes, you smoke _____ cigarette in most cases per day.

AL1. Have you used to drink? (>3 times per week for 6 consecutive months or more)

0···no   1···yes

AL2. If yes, you drink _____ milliliter in most cases per day.

AL3. What is your drinking type in most cases?

1···white wine　2···beer　3···red wine　4···wine　5···imported wine

6···other (Please indicate on the line:______)

Personal and family history of diseases

Put the corresponding number in the cell according to your situation

Diseases	Have you ever been diagnosed with the disease?<br>0···no 1···yes<br>9···forget	Diagnosis year	Have your immediate family ever been diagnosed with the disease?<br>0···no 1···yes 9···unclear	Relationship with you:<br>1···father 2···mother<br>3···sister or brother<br>4···child
Hypertension				
Diabetes				
Hyperlipidemia				
Fatty liver				
Liver cirrhosis				
Viral hepatitis				
Coronary disease				
Cholecystitis				
Stroke				
Cancer				
Mental illness				
