# Supplementary material for: A combined association of serum uric acid, alanine aminotransferase and waist circumference with non-alcoholic fatty liver disease: a community-based study
Source: PeerJ. 2022 Mar 4;10:e13022. doi: 10.7717/peerj.13022 (PMC8900609; doi:10.7717/peerj.13022)
Supplement: Supplemental Information 3 [file peerj-10-13022-s003.docx]

**Supplementary Table 1**

Comparison of characteristics according to SUA cutoff points

| **Variables** | **Normal SUA level** | **High SUA level** | ***P*** |
| --- | --- | --- | --- |
|  | **(n = 2739)** | **(n = 572)** |  |
| Gender (male), n (%) | 1911 (69.8) | 533 (93.2) | **< 0.001 ^a^** |
| Age (years) | 40.16 ± 9.33 | 39.70 ± 9.86 | 0.289 ^b^ |
| BMI (kg/m^2^) | 23.08 ± 2.87 | 25.12 ± 2.60 | **< 0.001 ^b^** |
| WC (cm) | 81.83 ± 9.35 | 88.44 ± 8.20 | **< 0.001 ^b^** |
| SBP (mmHg) | 124.42 ± 15.20 | 130.78 ± 14.23 | **< 0.001 ^b^** |
| DBP (mmHg) | 75.04 ± 10.27 | 79.61 ± 10.49 | **< 0.001 ^b^** |
| ALT (IU/L) ^†^ | 18.0 (13.0, 26.0) | 28.0 (18.0, 42.0) | **< 0.001 ^c^** |
| AST (IU/L) | 19.90 ± 7.62 | 23.72 ± 8.60 | **< 0.001 ^b^** |
| FPG (mmol/L) | 3.65 ± 1.62 | 3.65 ± 1.61 | 0.946 ^b^ |
| TG (mmol/L) ^†^ | 1.09 (0.80, 1.55) | 1.65 (1.13, 2.34) | **< 0.001 ^c^** |
| TC (mmol/L) | 4.54 ± 0.83 | 4.83 ± 0.90 | **< 0.001 ^b^** |

Abbreviations: *BMI* body mass index, *WC* waist circumference, *SBP* systolic blood pressure, *DBP* diastolic blood pressure, *ALT* alanine aminotransferase, *AST* aspartate aminotransferase, *FPG* fasting plasma glucose, *TG* triacylglycerol, *TC* total cholesterol.

^†^ non-normally distributed variables.

^a^ Chi square test; ^b^ independent-samples T-test; ^c^ Mann-Whitney U-test

Normal SUA level, ≤ 420 μmol/L in males and ≤ 360 μmol/L in females; High SUA level, > 420 μmol/L in males and > 360 μmol/L in females.

Results in bold type indicate statistically significant.

**Supplementary Table 2**

Comparison of characteristics according to ALT cutoff points

| **Variables** | **Normal ALT level** | **High ALT level** | ***P*** |
| --- | --- | --- | --- |
|  | **(n = 2897)** | **(n = 414)** |  |
| Gender (male), n (%) | 2057 (71.0) | 387 (93.5) | **< 0.001 ^a^** |
| Age (years) | 40.31 ± 9.47 | 38.44 ± 8.95 | **< 0.001 ^b^** |
| BMI (kg/m^2^) | 23.13 ± 2.82 | 25.58 ± 2.80 | **< 0.001 ^b^** |
| WC (cm) | 82.01 ± 9.22 | 89.82 ± 8.57 | **< 0.001 ^b^** |
| SBP (mmHg) | 124.64 ± 14.97 | 131.72 ± 15.60 | **< 0.001 ^b^** |
| DBP (mmHg) | 75.20 ± 10.25 | 80.19 ± 10.81 | **< 0.001 ^b^** |
| AST (IU/L) | 18.64 ± 4.46 | 33.99 ± 12.58 | **< 0.001 ^b^** |
| SUA (μmol/L) | 331.99 ± 81.32 | 398.49 ± 81.16 | **< 0.001 ^b^** |
| FPG (mmol/L) | 3.65 ± 1.59 | 3.64 ± 1.84 | 0.863 ^b^ |
| TG (mmol/L) ^†^ | 1.10 (0.81, 1.59) | 1.69 (1.19, 2.47) | **< 0.001 ^c^** |
| TC (mmol/L) | 4.55 ± 0.83 | 4.89 ± 0.93 | **< 0.001 ^b^** |

Abbreviations: *BMI* body mass index, *WC* waist circumference, *SBP* systolic blood pressure, *DBP* diastolic blood pressure, *AST* aspartate aminotransferase, *SUA* serum uric acid, *FPG* fasting plasma glucose, *TG* triacylglycerol, *TC* total cholesterol.

^†^ non-normally distributed variables.

^a^ Chi square test; ^b^ independent-samples T-test; ^c^ Mann-Whitney U-test

Normal ALT level, ≤ 40 IU/L; High ALT level, > 40 IU/L.

Results in bold type indicate statistically significant.

**Supplementary Table 3**

Comparison of characteristics according to WC cutoff points

| **Variables** | **Normal WC level** | **High WC level** | ***P*** |
| --- | --- | --- | --- |
|  | **(n = 2549)** | **(n = 679)** |  |
| Gender (male), n (%) | 1748 (68.6) | 625 (92.0) | **< 0.001 ^a^** |
| Age (years) | 39.84 ± 9.20 | 42.40 ± 9.47 | **< 0.001 ^b^** |
| BMI (kg/m^2^) | 22.53 ± 2.36 | 26.87 ± 2.29 | **< 0.001 ^b^** |
| SBP (mmHg) | 123.33 ± 14.52 | 133.65 ± 15.39 | **< 0.001 ^b^** |
| DBP (mmHg) | 74.49 ± 9.97 | 81.25 ± 10.51 | **< 0.001 ^b^** |
| ALT (IU/L) ^†^ | 17.0 (13.0, 25.0) | 28.0 (20.0, 42.0) | **< 0.001 ^c^** |
| AST (IU/L) | 19.69 ± 7.37 | 23.58 ± 8.78 | **< 0.001 ^b^** |
| SUA (μmol/L) | 327.38 ± 80.37 | 386.20 ± 82.37 | **< 0.001 ^b^** |
| FPG (mmol/L) | 3.58 ± 1.52 | 4.07 ± 1.93 | **< 0.001 ^b^** |
| TG (mmol/L) ^†^ | 1.07 (0.79, 1.50) | 1.66 (1.17, 2.32) | **< 0.001 ^c^** |
| TC (mmol/L) | 4.55 ± 0.82 | 4.79 ± 0.95 | **< 0.001 ^b^** |

Abbreviations: *BMI* body mass index, *WC* waist circumference, *SBP* systolic blood pressure, *DBP* diastolic blood pressure, *ALT* alanine aminotransferase, *AST* aspartate aminotransferase, *SUA* serum uric acid, *FPG* fasting plasma glucose, *TG* triacylglycerol, *TC* total cholesterol.

^†^ non-normally distributed variables.

^a^ Chi square test; ^b^ independent-samples T-test; ^c^ Mann-Whitney U-test

Normal WC level, ≤ 90 cm in males and ≤ 85 cm in females; High WC level, > 90 cm in males and > 85 cm in females.

Results in bold type indicate statistically significant.
